# Local root abscisic acid (ABA) accumulation depends on the spatial distribution of soil moisture in potato: implications for ABA signalling under heterogeneous soil drying

**DOI:** 10.1093/jxb/eru501

**Published:** 2014-12-29

**Authors:** Jaime Puértolas, María R. Conesa, Carlos Ballester, Ian C. Dodd

**Affiliations:** ^1^Lancaster Environment Centre, Lancaster University, Lancaster LA1 4YQ, UK; ^2^Escuela Técnica Superior de Ingeniería Agronómica, Universidad Politécnica de Cartagena, Paseo Alfonso XIII 48, Cartagena, Murcia 30203, Spain; ^3^Centro para el Desarrollo de la Agricultura Sostenible, Instituto Valenciano de Investigaciones Agrarias, CV 315, Km. 10.7, Moncada, Valencia 46113, Spain

**Keywords:** Partial root-zone drying, root ABA, root-to-shoot signalling, root water potential, root water uptake, stomatal conductance, water-saving irrigation.

## Abstract

Patterns of root abscisic acid (ABA) accumulation ([ABA]_root_), root water potential (Ψ_root_), and root water uptake (RWU), and their impact on xylem sap ABA concentration ([X-ABA]) were measured under vertical partial root-zone drying (VPRD, upper compartment dry, lower compartment wet) and horizontal partial root-zone drying (HPRD, two lateral compartments: one dry, the other wet) of potato (*Solanum tuberosum* L.). When water was withheld from the dry compartment for 0–10 d, RWU and Ψ_root_ were similarly lower in the dry compartment when soil volumetric water content dropped below 0.22cm^3^ cm^–3^ for both spatial distributions of soil moisture. However, [ABA]_root_ increased in response to decreasing Ψ_root_ in the dry compartment only for HPRD, resulting in much higher ABA accumulation than in VPRD. The position of the sampled roots (~4cm closer to the surface in the dry compartment of VPRD than in HPRD) might account for this difference, since older (upper) roots may accumulate less ABA in response to decreased Ψ_root_ than younger (deeper) roots. This would explain differences in root ABA accumulation patterns under vertical and horizontal soil moisture gradients reported in the literature. In our experiment, these differences in root ABA accumulation did not influence [X-ABA], since the RWU fraction (and thus ABA export to shoots) from the dry compartment dramatically decreased simultaneously with any increase in [ABA]_root_. Thus, HPRD might better trigger a long-distance ABA signal than VPRD under conditions allowing simultaneous high [ABA]_root_ and relatively high RWU fraction.

## Highlight

Local root abscisic acid (ABA) accumulation depends on the spatial distribution of soil moisture in potato. Implications for ABA signalling under heterogeneous soil drying are discussed.

## Introduction

Root-sourced abscisic acid (ABA) is considered a long-distance chemical signal that triggers physiological responses enhancing plant water economy (stomatal closure, decrease in leaf growth) in response to soil drying (Tardieu *et al.*, 1996; Zhang and Davies, 1990). When water is withheld, upper soil layers usually dry faster since they are exposed to evaporation and contain the highest root density, and thus soil moisture increases progressively with depth (e.g. Zhang and Davies, 1989; Beff *et al.*, 2013; Puértolas *et al.*, 2014). Moreover, some irrigation techniques, such as partial root-zone drying (PRD), intend to induce lateral soil moisture heterogeneity by applying irrigation only to part of the root system (Stoll *et al.*, 2000). Modelling of ABA signalling under heterogeneous soil moisture is essential to understand plant responses to soil drying under field conditions. This knowledge could inspire more efficient irrigation scheduling to maximize water-use efficiency (Dodd *et al*., 2008*a*, *b*; Liu *et al.*, 2008).

Studies on ‘one shoot–two root’ grafted plants with only one of the root systems subjected to soil drying have shown that root xylem sap ABA concentration coming from the ‘dry’ root increased compared with the ‘wet’ root, initially increasing xylem sap ABA concentration above the graft union. Later, as water uptake from the dry side decreased, xylem sap ABA concentration ([X-ABA]) subsequently decreased (Dodd *et al.*, 2008*a*), similar to observations in some PRD experiments (Khalil and Grace, 1993; Dry *et al.*, 2000). However, the response of [X-ABA] to PRD is not uniform, as some studies report no increase at all in ABA concentration in the xylem sap or in the leaf (Blackman and Davies, 1985; Wakrim *et al.*, 2005; Jensen *et al.*, 2009; Einhorn *et al.*, 2012). Further understanding of the factors causing these different responses to different soil moisture gradients is essential for modelling ABA signalling in response to soil drying.

Although 25–30% of the ABA in xylem sap might come from shoots due to recirculation of basipetally transported ABA in the phloem (Liang *et al.*, 1997), the initial increase in xylem ABA concentration in response to water stress is believed to be triggered mainly by increased root ABA accumulation (Zhang and Davies, 1990; Neales and McLeod, 1991). Therefore, understanding ABA accumulation patterns within the root system is essential to predict long-distance ABA signalling responses to heterogeneous soil drying. Root ABA concentration increases in response to soil drying due to an increase in ABA biosynthesis by roots and ABA recirculation from shoots via phloem transport (Sauter *et al.*, 2001). However, there are few studies on the effect of soil moisture heterogeneity on the spatial distribution of root ABA accumulation. Local soil moisture and root ABA concentration were tightly correlated under PRD in a split-root experiment in sycamore (Khalil and Grace, 1993). When maize was grown in drying soil columns, root ABA concentrations increased in drying soil layers, but continued soil drying later decreased root ABA concentrations (Zhang and Davies, 1989). In contrast, there was no clear relationship between root ABA concentration and local soil moisture in bean growing in drying soil columns (Trejo and Davies, 1991; Puértolas *et al.*, 2013). Understanding the origin of these discrepancies is essential to predict xylem ABA concentration based on differences in local soil moisture.

ABA synthesis in root cells increases as root water potential (Ψ_root_) decreases (Zhang and Davies, 1987; Simonneau *et al.*, 1998). Therefore, heterogeneity in Ψ_root_ throughout the root system might be the main factor explaining local differences in root ABA accumulation when soil moisture is distributed heterogeneously. Thus, for the same level of soil drying, ABA export from root to shoots could differ according to how soil moisture distribution affects Ψ_root_ within the root zone.

If this hypothesis is confirmed, understanding how Ψ_root_ is distributed across the root zone in response to soil moisture heterogeneity is essential to understand ABA signalling responses to soil drying. Some studies have shown differences in Ψ_root_ across the root zone when soil moisture is heterogeneous (Dodd *et al*., 2008*a*; Simonneau and Habib, 1994), but other authors have also reported that, because of this water potential gradient, water can be redistributed within the plant in general and the root system in particular (de Kroon *et al.*, 1996; Stoll *et al.*, 2000). Therefore, the existence and extent of Ψ_root_ heterogeneity across the root zone could be modulated by water redistribution and differences in hydraulic resistance within the root zone. Although water movement within the root system depends on water and osmotic potential gradients (Mao *et al.*, 2009), it is plausible that vertical water recirculation might be more effective than lateral recirculation, as water transport through the vessels via the xylem might be more effective than cell-to-cell pathways (Steudle and Peterson, 1998). This could explain the observed homogeneity of Ψ_root_ under vertical soil moisture gradients (Puértolas *et al.*, 2013), unlike in split-root experiments subjected to lateral gradients (Simonneau and Habib, 1994; Dodd *et al.*, 2008*a*). Equilibration of water potential within the root zone required night-time cessation of transpiration (Bauerle *et al.*, 2008). This also suggests the possibility of diurnal fluctuations in water potential heterogeneity, which might influence root ABA accumulation and, in turn, root-to-shoot ABA signalling responses to soil moisture heterogeneity.

This study aimed to determine the relationship of local soil moisture with Ψ_root_, ABA concentration, and water uptake when soil moisture is heterogeneously distributed, and the influence of these variables on ABA export to the shoot. We assessed the following hypotheses: (i) ABA accumulation is higher in roots growing in drying soil than in wet soil; (ii) differences in Ψ_root_ are associated with differential ABA accumulation within the root system, and are lower under vertical soil moisture gradients than under lateral gradients; and (ii) root water potential and ABA accumulation gradients within the root zone change during the day, with lower heterogeneity during night-time cessation of transpiration. To assess these hypotheses, root ABA concentrations ([ABA]_root_) and Ψ_root_ were measured in hydraulically isolated soil compartments at progressive degrees of soil drying when soil moisture heterogeneity was imposed laterally or vertically. The experiments used potato, since its ABA signalling has previously been investigated under lateral PRD (Liu *et al.*, 2008), yet there are pronounced vertical gradients in soil moisture under field-grown potato crops even with the application of PRD (Puértolas *et al.*, 2014). Understanding ABA signalling in the field therefore requires knowledge on how vertical and lateral soil moisture gradients affect root ABA accumulation.

## Material and methods

### Experiment 1: vertical PRD (VPRD) in soil columns

Potato seed tubers (*Solanum tuberosum* L. cv. ‘Maris Piper’) were sown in pots filled with a fertilized organic loam (John Innes No. 2; J. Arthur Bowers, UK). A moisture release curve for this substrate can be found in Puértolas *et al.* (2013). Pots were cylinders, 6.5cm in diameter and 23cm in length (0.75cm^3^ in volume), with stainless steel mesh (0.7mm aperture) at the base to assist drainage, and were designed to fit tightly in a pressure chamber of the same volume (Soil Moisture Equipment Corp., Santa Barbara, CA, USA). A 1.5cm thick quartzite gravel (15–20mm) layer divided the upper from the lower half of the pot, preventing water ascent by capillarity from the lower to the upper part. Before filling, pots were cut in half lengthwise and the two halves were stuck together with duct tape to allow easy extraction of the intact soil column at harvest. Two pairs of 6mm diameter holes (2cm apart) were punched in the wall of the pot, at the midpoint of each pot compartment. Each pair of holes allowed insertion of the two rods of a soil moisture capacitance probe (Model SM-200; Delta-T, Burwell, UK) to estimate root water uptake (RWU) from each soil layer. Plants were placed in a controlled environment room, providing 400–600 μmol m^–2^ s^–1^ of irradiance, 14h of photoperiod, and 24/19 °C day/night temperature. Five batches of 11 plants were sown every other day (55 plants in total were measured). All plants in each batch were measured within 2 d.

Plants were harvested 4 weeks after emergence. All plants were watered to field capacity in the morning by completely immersing the pots in water. Plants were randomly assigned to four groups, which were subjected to 0, 1, 2, or 3 d of VPRD. During PRD, only the bottom half of the pot was immersed once a day. Plants with 0 d of VPRD application were fully irrigated until the morning of their measurement date. Each treatment comprised 13–14 plants.

**Fig. 1.**
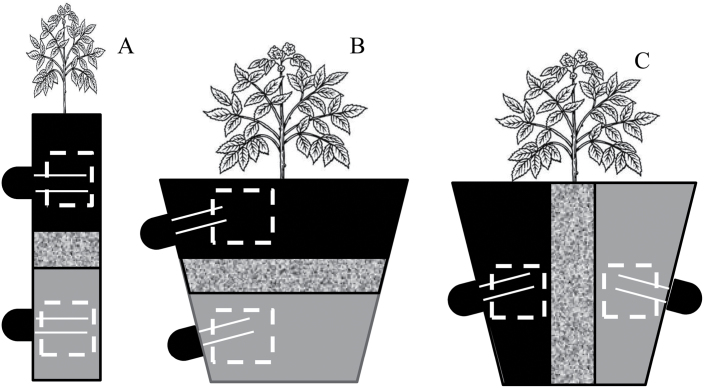
Schematic representation of the experimental application of partial root-zone drying in Experiment 1 (A), and Experiment 2 for vertical PRD (B), horizontal PRD (C). Grey areas represent wet compartments and black areas dry compartments. The central grainy stripe represents a gravel layer separating the wet and dry compartments. The locations of SM-200 soil moisture probes (black semi-circles) and the approximate location of root sampling (white dashed square) are also depicted. The dimensions of the two different pots, the sensor rods and the gravel layer are depicted to scale.

Measurements were taken 4–8h after the lights were switched on in the controlled environment room. Before the measurements, the pot surface and base were covered with duct tape to minimize soil evaporation, weighed on a precision balance to 0.01g (Adventurer Pro AV4102; Ohaus, Thetford, UK) and soil moisture sensors (Model SM200; Delta-T) inserted in each pair of holes (Fig. 1). After 1.5–2h, the sensors were removed, the pot weighed again, and stomatal conductance (*g*_s_) was measured in three mature leaflets with a porometer (AP4; Delta-T). Whole-plant water uptake was calculated from the initial and final pot weights. Evaporation was assessed by determining the water loss from a well-watered pot (without a plant) and ignored as negligible (<3% of the water loss of pots containing a plant). Water uptake from each compartment was calculated from soil moisture sensor readings as described by Puértolas *et al*. (2013) Soil volumetric water content (*θ*_v_) in each compartment at the time of measurement was also recorded for each plant. One of the leaves in which stomatal conductance was measured was excised and leaf water potential (Ψ_leaf_) was determined with a pressure chamber. The plant was then de-topped and the pot inserted in a pressure chamber with the stem protruding to determine Ψ_root_. Since sap flow rate can influence [X-ABA] (Dodd *et al.*, 2008*a*), sap was collected at different overpressures above the balance pressure (0.4, 0.6, and 0.8MPa) as described previously (Dodd *et al.*, 2008*a*). Sap was collected in pre-weighed Eppendorf vials, frozen in liquid nitrogen immediately after being collected, and stored at –18 °C for ABA determination. Sap flow rate generated at each overpressure was calculated by weighing the vials before freezing them and recording the elapsed collection time. ABA concentration was determined in the sample with the closest sap flow rate to the actual transpirational flow rate (calculated by weighing the pot).

Each pot was opened and a root segment (3–6mg in root dry weight) from the interior of each column compartment was excised, briefly (<10 s) washed in tap water to remove adhering soil particles, frozen in liquid nitrogen, and stored at –18 °C for ABA determination. The elapsed time between excision and freezing did not exceed 20 s.

Root samples for ABA determination were freeze dried and finely ground. Deionized water was added at 1:50 weight ratio. Both sap samples and root extracts were analysed by a radioimmunoassay (Quarrie *et al.*, 1988) to obtain root xylem sap ABA concentration ([X-ABA]_root_) and root ABA concentration ([ABA]_root_). [X-ABA]_root_ was measured in sap samples, while [ABA]_root_ was measured in the aqueous extract obtained after incubating freeze-dried root tissue samples in a shaker at 4 °C overnight. Xylem sap of *S. tuberosum* does not present non-specific interference in the assay (Liu *et al.*, 2006). No cross-reaction of the antibody with other compounds in root aqueous extracts was detected, based on a cross-reactivity test (Quarrie *et al.*, 1988). [X-ABA]_root_ decreased as collecting pressure increased (*F*=30.8, *P*<0.001).

Roots were extracted, oven dried, and weighed. No differences between the upper and bottom sides in root dry weight were observed (data not shown).

### Experiment 2: VPRD vs horizontal PRD(HPRD)

The experiment was repeated twice. On each occasion, 26 potato seed tubers (*S. tuberosum* L. cv. ‘Horizon’) were sown in 0.25 l pots filled with the same soil used in Experiment 1. Plants were transplanted 5–7 d after emergence into 5 l pots (17.5cm in height, 22.5cm in upper diameter) divided into two compartments with equal capacity by a 3–4cm layer of the same gravel type as in Experiment 1 to disrupt water capillarity between compartments. Half of the pots were divided by a vertical gravel layer (producing two lateral compartments of 1800cm^3^ capacity; HPRD). The rest of the pots were divided by a horizontal layer (producing two vertical compartments of ~1800cm^3^ capacity; VPRD) (Fig. 1). In HPRD pots, a 5×5cm acetate window was opened in the pot wall at mid-height on each side to allow root sampling, while in VPRD there was a single 14cm high×5cm wide window, which spanned the two vertical sides of the pot. Additionally, a pair of holes was punched in each compartment to insert SM-200 probes. In HPRD, each pair of holes was placed in the middle of the wall beside each opposite window, while in VPRD each pair of holes was located in the middle of each vertical compartment and close to the window. Windows were covered with the same cut piece of pot wall and fixed with duct tape until sampling.

Plants were grown for 5 weeks after emergence, before measurement under the same environmental conditions described in Experiment 1. They were watered to field capacity after transplanting until PRD was applied. For each plant, PRD was applied for a varying period of time before measurement (from 0 to 9 d) to obtain a wide range of soil moisture content on the dry side. During PRD application, plants were watered once a day. In HPRD, pots were inclined 20° and irrigation (to field capacity) was applied to the lower side of the pot to prevent water running through the layer towards the dry side. After 15min, pots were placed back in the normal position. In VPRD, the bottom half of the pot was immersed in water for 5 s as in Experiment 1.

Half of the plants were measured at the end of the night period (pre-dawn measurements), and the rest between 6 and 8h after the beginning of the photoperiod (midday measurements). Two or three plants were measured per day and time of day during a 7 d period. At midday, water uptake from the two compartments was measured as described in Experiment 1.

At both times of the day, a 2cm root segment was excised from the edge of the pot after partially cutting the acetate window as described by Bauerle *et al.* (2008). Roots were sampled around the centre of each window in HPRD (at ~8.5cm from the soil surface) and at the midpoint in each vertical compartment in VPRD (at ~4cm from the soil surface and the bottom of the pot for upper and lower compartments, respectively). The root segment was tapped to remove adhering soil particles, briefly blotted with absorbent paper, and immediately placed and sealed inside a psychrometric chamber (C52; Wescor, Logan, UT, USA). From excision to sealing inside the chamber, the elapsed time was less than 10 s. Two samples were taken from each plant, one per compartment. The chamber was placed in a room at stable temperature and humidity conditions. Root water potential (Ψ_root_) was measured after thermal equilibration by dew point psychrometry with a microvoltmeter (HR33-T; Wescor). Preliminary tests determined that thermal equilibration was attained after at least 6h. Each chamber was calibrated previously with NaCl solutions of known water potential. Another root sample was excised through the opening in the wall and processed as in Experiment 1 to determine [ABA]_root_.

For midday measurements, stomatal conductance was measured in three fully expanded leaves located in the upper third of the canopy. For pre-dawn and midday measurements, Ψ_leaf_ and leaf xylem sap ABA concentration ([X-ABA]_leaf_) were determined as described for Experiment 1.

*θ*_v_ and RWU were measured at midday with the soil moisture sensors as described in Experiment 1. For both pre-dawn and midday plants, a sample of soil from each compartment was weighed, dried at 80 °C for 4 d and the soil gravimetric water content (*θ*_g_) was determined. A tight linear relationship between *θ*_v_ and *θ*_g_ was observed in midday plants [*θ*_g_ (g g^–1^)=0.94×θ_v_ (cm^3^ cm^–3^)+0.02; *r* ^2^=0.91; data not shown], which was used to convert *θ*_g_ into *θ*_v_ for all plants to allow comparison with previous studies (e.g. Dodd *et al*., 2008*a*, *b*; Liu *et al.*, 2008; Puértolas *et al.*, 2013).

No differences between wet and dry sides in root dry weight were observed in any of the two PRD distributions (data not shown).

### Statistical analyses

No statistical differences in any of the studied variables were found between batches of plants (five batches in Experiment 1, two batches in Experiment 2), so all the plants were pooled in both experiments.

Visual inspection of the relationships between soil moisture and different variables, especially [ABA]_root_, revealed that altered physiological responses were only observed in plants with *θ*_v_ <0.22cm^3^ cm^–3^ in the dry compartment. Plants then were separated into two groups based on *θ*_v_ in the dry compartment: dry, *θ*_v_<0.22cm^3^ cm^–3^; wet, *θ*_v_>0.22cm^3^ cm^–3^. Those groups were used to assess the effect of soil moisture in the dry compartment on [ABA]_root_, RWU, and Ψ_root_ by repeated-measures analysis of variance (ANOVA) with PRD side (wet or dry) as the within factor (S) and dry compartment soil moisture (D) as the between factor in Experiment 1. For Experiment 2, S was considered the within factor, and D, measurement time of the day (T), and PRD spatial distribution (P – VPRD or HPRD) were the between factors. [X-ABA], Ψ_leaf_, and g_s_ were assessed by ANOVA with D as the fixed factor in Experiment 1. For Experiment 2, T and P were the fixed factors. Relationships between different variables were assessed by linear regression. All statistical analyses were carried out with SPSS 20 (IBM, Armonk, NY, USA).

## Results

### Local root responses

**Fig. 2.**
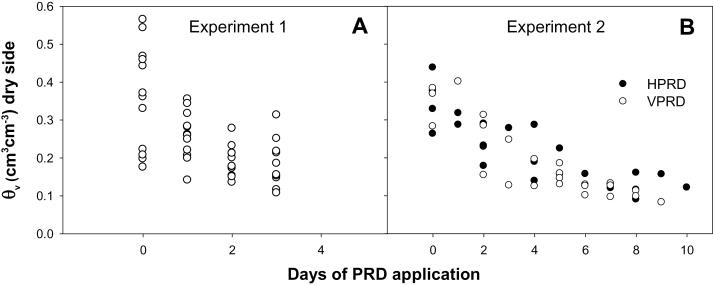
Measurement of *θ*_v_ in the dry compartment under different lengths of PRD application in Experiment 1 (A) and each PRD distribution (HPRD and VPRD) in Experiment 2 (B). Each point is an individual root system.

*θ*_v_ was lower in the dry compartment, and decreased with time of water withholding in both experiments, although it was faster in the small columns of Experiment 1 (Fig. 2). Average soil water content in the dry side decreased with time of PRD application to ~0.10cm^3^ cm^–3^ in Experiment 2, but only to 0.19cm^3^ cm^–3^ in Experiment 1, although some individuals were close to 0.10cm^3^ cm^–3^ (Fig. 2). Daily irrigation of the wet side maintained *θ*_v_ generally above 0.30cm^3^ cm^–3^ (data not shown) for both experiments. In Experiment 2, average (whole-pot) soil moisture (mean of wet and dry sides) was never below 0.16cm^3^ cm^–3^. PRD distribution did not influence *θ*_v_, as it was similarly lower in the dry side for both HPRD and VPRD.

**Fig. 3.**
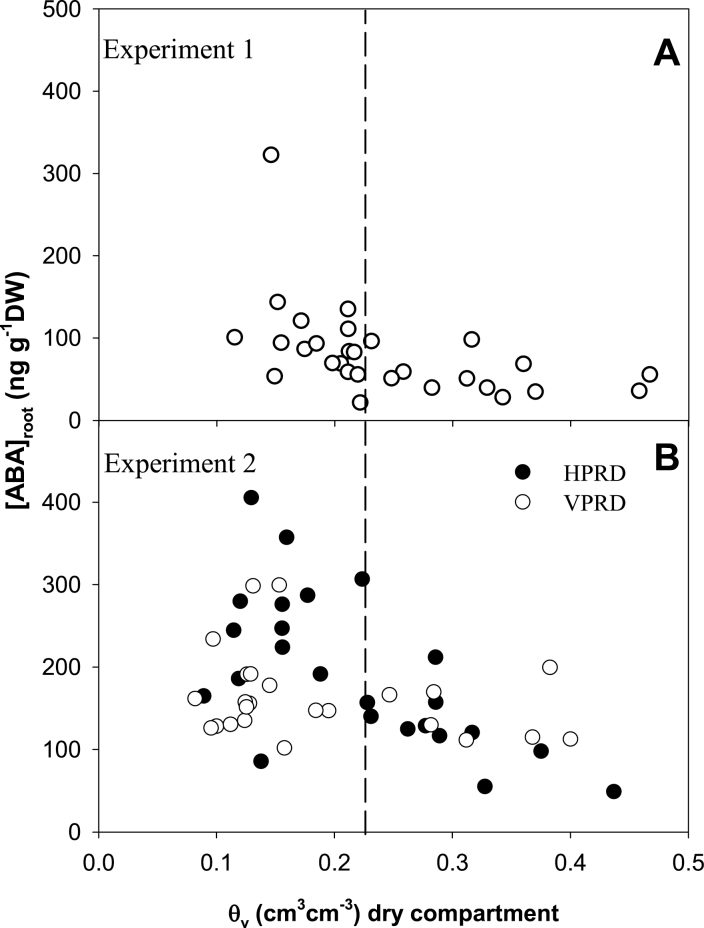
Relationship between θ_v_ in the dry compartment and [ABA]_root_ for Experiment 1 (A) and each PRD distribution (HPRD and VPRD) in Experiment 2 (B). Since no differences between pre-dawn and midday were found, data was pooled for Experiment 2. The dashed line is drawn at *θ*_v_=0.22cm^3^ cm^–3^. Each point is an individual root system.

**Fig. 4.**
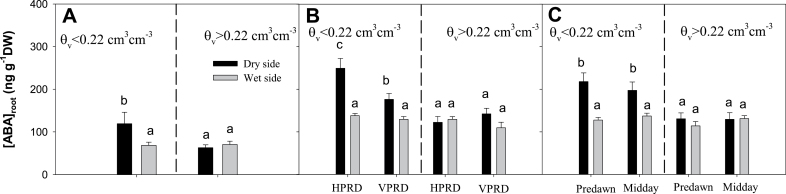
[ABA]_root_ [mean±standard error (SE)] in both compartments in plants with soil volumetric moisture in the dry compartment below (left of the dashed line dividing each panel) and above (right) 0.22cm^3^ cm^–3^. (A) Experiment 1. (B) Experiment 2: HPRD versus VPRD (both times of the day pooled). (C) Experiment 2: pre-dawn versus midday measurements (both PRD distributions pooled). Different letters denote statistical differences across compartments and groups within the same panel.

[ABA]_root_ in the dry compartment increased but only when *θ*_v_ was below 0.22cm^3^ cm^–3^ in both experiments (Figs 3 and 4). It did not increase in the wet side regardless of the soil moisture in the dry side (Fig. 4). In Experiment 2, this increase in the dry compartment was higher in HPRD than VPRD, despite statistically similar *θ*_v_ (Fig. 4, *P*=0.008 for the compartment×soil moisture level×PRD distribution interaction; Table 1). However, no interaction with time of the day (pre-dawn versus midday) was detected (Fig. 4, *P*=0.78 for the side×soil moisture level×time of the day interaction).

**Table 1.** Repeated-measures ANOVA of variables measured in the two compartments of the three different experimentsStatistically significant effects are indicated in bold. NA, not applicable.

	[ABA]_root_			RWU		Ψ_root_	
	D.f.	F	P	F	P	F	P
**Experiment 1**							
Compartment (C)	1	0.4	0.56	35	**<0.001**	NA	NA
Dry compartment moisture (D)	1	2.3	0.14	17	**<0.001**	NA	NA
C×D	1	6.5	**0.02**	17	**<0.001**	NA	NA
**Experiment 2**							
Compartment (C)	1	21	**<0.001**	27	**<0.001**	12	**<0.001**
PRD distribution (P)	1	2.0	0.18	2.0	0.18	2.7	0.11
Time of the day (T)	1	0.0	0.97	NA	NA	0.3	0.60
Dry compartment moisture (D)	1	13	**<0.001**	3.1	0.09	10	**0.004**
C×P	1	0.3	0.62	0.4	0.53	0.1	0.81
C×T	1	0.9	0.36	NA	NA	0.5	0.50
C×D	1	8.2	**0.006**	24	**<0.001**	13	**<0.001**
P×T	1	0.2	0.64	NA	NA	0.1	0.74
P×D	1	5.6	**0.02**	3.8	0.07	0.0	0.98
T×D	1	0.1	0.77	NA	NA	2.6	0.12
C×P×T	1	0.1	0.78	NA	NA	0.0	0.94
C×P×D	1	7.6	**0.008**	1.5	0.24	0.0	0.94
P×T×D	1	0.4	0.55	NA	NA	0.1	0.80
C×T×D	1	0.1	0.79	NA	NA	0.3	0.58

**Fig. 5.**
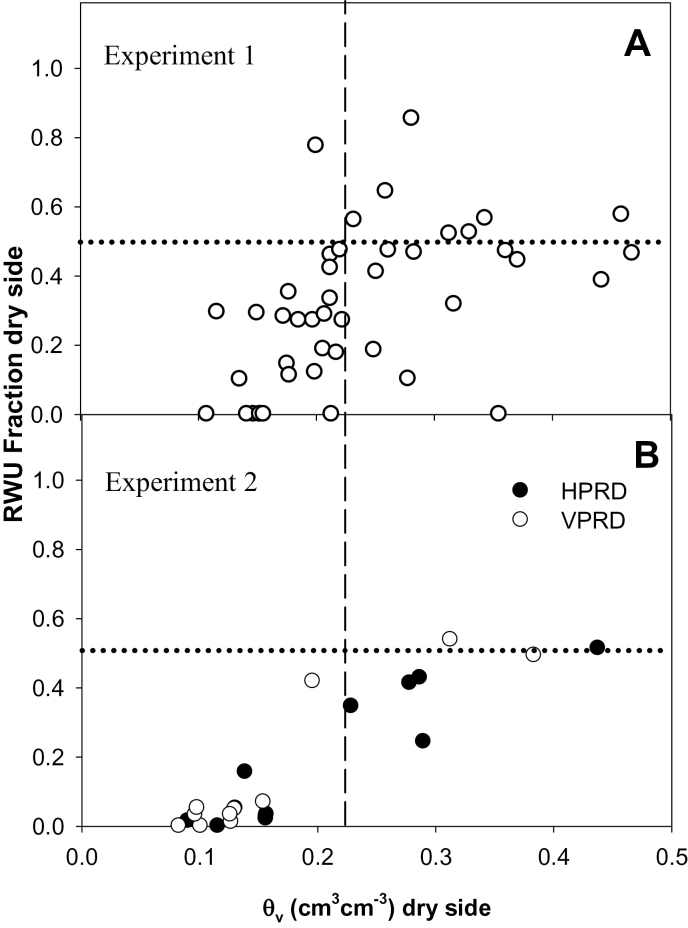
Relationship between *θ*_v_ in the dry compartment and RWUF for Experiment 1 (A) and each PRD distribution (HPRD and VPRD) in Experiment 2 (B). The dashed vertical line is drawn at *θ*_v_=0.22cm^3^ cm^–3^; the dotted horizontal line is at RWUF=0.5. Each point is an individual root system.

**Fig. 6.**
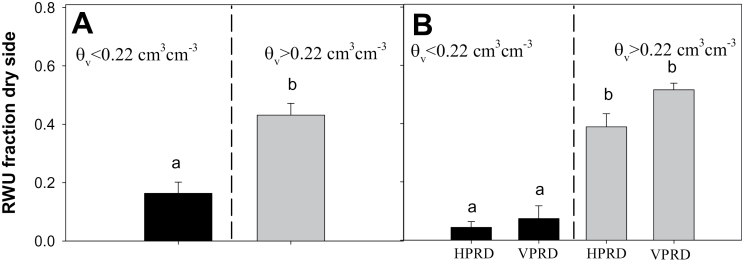
RWUF in the dry compartment (mean±SE) in plants with soil volumetric moisture in the dry compartment below (left of the dashed line dividing each panel) and above (right) 0.22cm^3^ cm^–3^. (A) Experiment 1. (B) Experiment 2: HPRD versus VPRD. Different letters denote statistical differences across compartments and groups within the same panel.

RWU was much higher in the wet compartment (Table 1). The fraction of RWU (RWUF) from the dry compartment to total uptake was positively related to soil moisture in the dry side in both experiments (Fig. 5), but in Experiment 2 it was much lower in plants when the dry compartment had lower soil moisture (*θ*_v_ <0.22cm^3^ cm^–3^; Fig. 6).

**Fig. 7.**
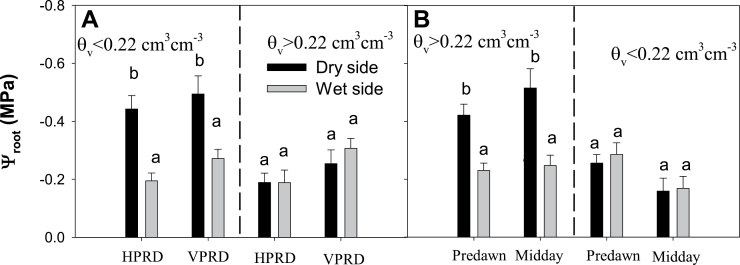
Ψ_root_ (mean±SE) in both compartments in plants from Experiment 2 with soil volumetric moisture in the dry compartment below (left of the dashed line dividing each panel) and above (right) 0.22cm^3^ cm^–3^. (A) HPRD versus VPRD (times of the day pooled). (b) Pre-dawn versus midday measurements (PRD distributions pooled). Different letters denote statistical differences across compartments and groups within the same panel.

**Fig. 8.**
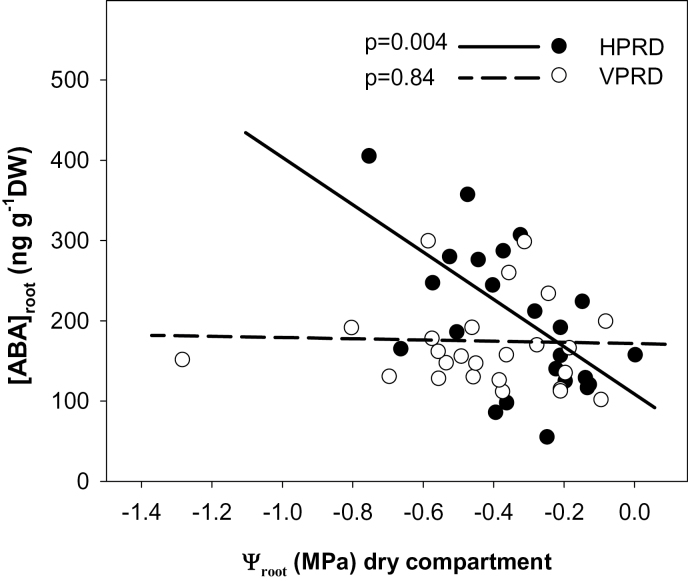
Relationship between Ψ_root_ and [ABA]_root_ in Experiment 2 for both PRD distributions. Regression lines and *P* values are shown for each PRD distribution. Each point is an individual root system.

Ψ_root_ in Experiment 2 was significantly lower in the dry side but only when soil moisture decreased below 0.22cm^3^ cm^–3^ (Table 1, Fig. 7). No effects of PRD distribution or time of the day were found. [ABA]_root_ in the dry side significantly increased as Ψ_root_ decreased in HPRD but not in VPRD (Fig. 8).

### Whole-plant responses

**Fig. 9.**
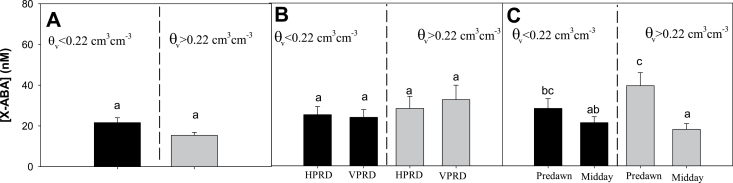
[X-ABA] (mean±SE) in plants with soil volumetric moisture in the dry compartment below (left of the dashed line dividing each panel) and above (right) 0.22cm^3^ cm^–3^. (A) Experiment 1. (B) Experiment 2: HPRD versus VPRD (times of the day pooled). (C) Experiment 2: pre-dawn versus midday measurements (PRD distributions pooled). For Experiment 1, [X-ABA] was determined in root xylem sap, while in Experiment 2 it was in the leaf. Different letters denote statistical differences across compartments and groups within the same panel.

[X-ABA]_root_ did not change with soil drying in Experiment 1 (Fig. 9). In Experiment 2, [X-ABA]_leaf_ was higher at pre-dawn, but VPRD and HPRD distributions had similar effects on [X-ABA]_leaf._

Ψ_leaf_ did not change with soil drying in any of the experiments (Table 2). It decreased by ~0.2MPa from pre-dawn to midday in Experiment 2 (Table 2; Ψ_leaf_=–0.21±0.01 and –0.45±0.02MPa for pre-dawn and midday, respectively), but there were no differences between PRD treatments. In Experiment 1, root xylem water potential did not differ significantly with different soil moisture in the dry compartment (*F*=1.2; *P*=0.25)

**Table 2.** ANOVA of variables measured in the whole plant of the three different experimentsSignificant effects are highlighted in bold. NA, not applicable.

	[X-ABA]			Ψ_leaf_		g_s_	
	d.f.	F	P	F	P	F	P
**Experiment 1**							
Dry compartment moisture (D)	1	0.3	0.75	1.9	0.16	0.1	0.95
**Experiment 2**							
PRD distribution (P)	1	3.4	0.07	0.3	0.60	0.0	0.96
Time of the day (T)	1	0.4	0.56	104	**<0.001**	NA	NA
Drying compartment moisture (D)	1	8.2	**0.006**	2.6	0.11	0.0	0.94
P×T	1	0.7	0.42	0.2	0.69	NA	NA
P×D	1	0.1	0.82	0.0	0.95	0.1	0.74
T×D	1	5.4	0.03	0.3	0.61	NA	NA
P×T×D	1	0.5	0.48	0.0	0.89	NA	NA

The value of *g*_s_ did not change across treatments or soil moisture levels in either experiment (Table 2).

## Discussion

When compared against conventional deficit irrigation where water is applied homogeneously to the entire root zone, agronomical responses to PRD applied under field conditions are far from uniform (Dodd, 2009). Differences in root hormonal responses to soil moisture heterogeneity may partially cause this inconsistency. Our results indicated that the spatial distribution of soil moisture heterogeneity (whether vertical or horizontal) influences the impact of local soil moisture conditions on root ABA accumulation, which can influence not only root-to-shoot ABA signalling but other plant responses like root growth (Sharp and LeNoble, 2002) or root hydraulic conductivity (Hose *et al.*, 2000). Although the roots sampled under VPRD or HPRD treatments were morphologically indistinguishable (despite being sampled at different depths in the soil profile), there were clear differences in their sensitivity of root ABA accumulation to Ψ_root_ (Fig. 8). Nevertheless, these differences in local root ABA accumulation had minimal impacts on root-to-shoot ABA signalling (Fig. 9), since root ABA accumulation was coincident with limited water uptake (and thus sap flow) from roots in drying soil (Fig. 6).

Root ABA accumulation was higher in the dry part of the root system coincident with lower Ψ_root_ in both experiments and PRD distributions (Fig. 4), consistent with the previously reported dependence of ABA synthesis on Ψ_root_ (Simonneau *et al.*, 1998). However, the increase in ABA accumulation with decreasing soil moisture and Ψ_root_ was much higher in HPRD (Figs 3 and 8), which suggests that the capacity of roots to accumulate ABA could depend on their position, as roots in the dry side of VPRD were sampled from a higher position in the soil profile than in HPRD. Greater root maturity in upper soil layers may explain the lower response of [ABA]_root_ to soil water potential observed in VPRD than in HPRD, as it has been reported that maize primary young roots and primary root tips start to accumulate ABA in response to soil drying at higher relative root water content than mature primary roots and secondary roots (Zhang and Tardieu, 1996). This explanation agrees with previous observations of root ABA accumulation in response to soil drying. While root ABA concentration was much greater in the dry side of sycamore seedlings growing in split pots (Khalil and Grace, 1993), it was not clearly higher in the upper layers of drying soil columns in bean (Trejo and Davies, 1991; Puértolas *et al.*, 2013). In an extreme case, root ABA accumulation in the upper soil layers of maize growing in 1 m columns was lower than at intermediate depth, despite higher soil water content in the latter (Zhang and Davies, 1989). All these observations seem to support the hypothesis that the capacity of roots to accumulate ABA in response to soil drying decreases in the upper older roots compared with deeper younger roots. To our knowledge, these results are the first evidence of different sensitivity of root ABA accumulation to changes in Ψ_root_ depending on root position in the root zone. This might explain the discrepancies in local root ABA accumulation responses to soil drying described above. Although the evidence found in this experiment strongly suggest the hypothesis of different sensitivity to water potential according to position within the root, this should be proven in an specific study, preferably in longer pots and with different species.

Equivalent Ψ_root_ in the dry compartment in both HPRD and VPRD treatments (at equivalent *θ*_v_) in Experiment 2 (Fig. 7, Table 1) suggests that internal hydraulic redistribution is equally effective, regardless of how soil moisture heterogeneity is imposed. Although hydraulic redistribution should occur via night-time water potential equilibration (Bauerle *et al.*, 2008), differences in Ψ_root_ between dry and wet compartments occurred both pre-dawn and at midday (Fig. 7), indicating that the Ψ_root_ disequilibrium was stable throughout the photoperiod. Therefore, root ABA accumulation seems to be affected only by the degree of soil drying, regardless of the spatial layout of soil moisture heterogeneity, and not by differential internal water redistribution.

The differential root ABA accumulation in drying soil depending on their position (or age) observed in Experiment 2 might affect [X-ABA], with potentially higher export in HPRD than in VPRD. However, no ABA differences between different soil moisture distributions were observed, as soil drying did not significantly affect [X-ABA]_leaf_ (Fig. 9). Increased [X-ABA]_leaf_, observed only at pre-dawn in plants with high soil moisture (Fig. 9), was not associated with increased [ABA]_root_ or decreased soil moisture in the dry compartment, and probably reflects a concentration effect due to low sap flow observed during the night period, as seen previously (Sobeih *et al.*, 2004).

**Fig. 10.**
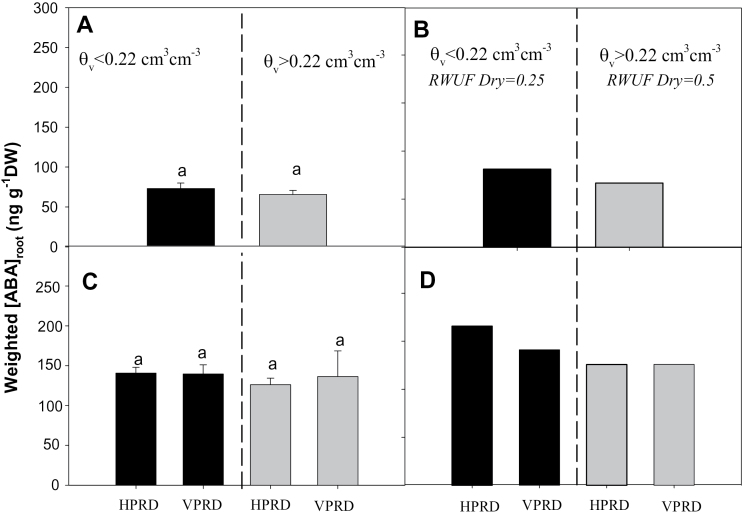
(A, C) Average [ABA]_root_ in both compartments weighted by RWUF [weighted [ABA]_root_=(RWUF_dry_×[ABA]_root dry_)+(RWUF_wet_×[ABA]_root wet_) (mean±SE)] in plants with soil volumetric moisture in the dry compartment above (left of the dashed line dividing each panel) and below (right) 0.22cm^3^ cm^–3^. (A) Experiment 1. (C) HPRD versus VPRD in Experiment 2. Different letters denote statistical differences across compartments and groups within the same panel. (B, D) Predicted weighted [ABA]_root_ assuming the observed [ABA]_root_ average in each side, level of soil moisture, and PRD distribution, but with RWUF in the dry side equal to 0.5 when *θ*_v_ >0.22, and RWUF in dry side equal to 0.25 when *θ*_v_ <0.22 in Experiment 1 (B) and for HPRD versus VPRD in Experiment 2 (D). Error bars are not applicable in (B) and (D) as they use modelled data.

This absence of an [X-ABA] response to PRD (Fig. 9) contrasts with results reported elsewhere (Sobeih *et al.*, 2004; Liu *et al.*, 2006; Wang *et al.*, 2010). However, the response of ABA status to PRD is not consistent, as some studies report no increase in xylem sap (Jensen *et al.*, 2009) or leaf (Blackman and Davies, 1985; Wakrim *et al.*, 2005; Einhorn *et al.*, 2012) ABA concentration. The lack of response of [X-ABA] to PRD observed (Fig. 9) seems to agree with a model predicting that [X-ABA] under PRD is determined by the average of root ABA in each side weighted by RWUF (Dodd *et al.*, 2008*a*) (Fig. 10A, C). In the two experiments described (Fig. 1), even though ABA accumulates in roots in the dry compartment, [X-ABA] cannot increase in response to PRD since the increase in [ABA]_root_ in the dry compartment is coincident with very low RWUF (<15%, Figs 4 and 6). Nevertheless, [X-ABA] of potato can increase in response to PRD (Liu *et al*., 2006, 2008). Although these studies did not simultaneously measure RWU and [ABA]_root_ during PRD, [X-ABA] increased (compared with fully irrigated plants) 2–4 d after applying PRD, when RWUF from the dry side exceeded 30% (Liu *et al.*, 2008). When RWU fell below 20% on d 5, [X-ABA] fell accordingly. These observations suggest that when soil moisture in the wet side is close to saturation, ABA signalling can only increase when experimental conditions simultaneously allow appreciable RWUF (at least 20%) and ABA accumulation in the dry compartment.

From a (PRD) management perspective, it becomes critical to determine experimental conditions that allow simultaneous root ABA accumulation and moderate water uptake from the dry side. The main difference between our study and previous studies in potato (Liu *et al*., 2006, 2008) is soil texture. Relationships between soil and root water potential, and *θ*_v_ and either root ABA levels or RWUF from drying roots under PRD conditions differ largely according to soil texture (Dodd *et al.*, 2010). While RWU declines more sensitively with *θ*_v_ in the organic loam used here than in a sandy soil (see Fig. 3a in Dodd *et al.*, 2010), both substrates showed similar increases in [X-ABA]_root_ as *θ*_v_ declined. Thus, similar root ABA accumulation would allow greater export to the shoots in a sandy soil than in the soil used in our study, thereby increasing [X-ABA] (Fig. 10). The reasons for higher RWUF from dry sand compartments are not clear but may depend on the soil water potential of the wet side: lower soil matric potential in the wet compartment attenuates the decrease in RWUF from the dry compartment (Dodd *et al.*, 2008*b*). Low matric potential in the wet side of sand-grown plants may be related to the difficulty of maintaining high matric potential in the wet compartment, as transpirational flow increases to balance the decrease in transpiration sourced from the dry compartment.

Our findings suggest that VPRD is less likely to increase [X-ABA] than HPRD, as [ABA]_root_ did not respond to decreasing Ψ_root_ in the former (Fig. 8). With a more moderate decrease in RWUF from the dry side with soil moisture than that observed (RWUF=0.25 when *θ*_v_ <0.22), while maintaining the observed [ABA]_root_ values in each side, the predicted weighted [ABA]_root_ average would only clearly increase in the HPRD treatment of Experiment 2 (Fig. 10B, D). This implies that transient increases in [X-ABA] in response to heterogeneous soil drying would only be significant when soil drying affects parts of the root system that are better able to synthesize ABA in response to decreasing Ψ_root_.

The relative insensitivity of ABA synthesis to soil drying in some parts of the root system (Fig. 3A) should be considered in designing more efficient water-saving irrigation strategies. For instance, VPRD application could be intentionally achieved using simultaneously subsurface and surface drip irrigation, keeping the lower layers permanently wet while submitting the upper layers to drying and rewetting cycles (Kang *et al.*, 2002). This may be a more efficient technique than HPRD, since the dry part of the root system would be the upper part, which produces less ABA (Fig. 4), maintaining higher stomatal conductance (and photosynthesis) than HPRD for a similar degree of soil drying (and irrigation volume application). Identifying the spatial patterns of local root ABA accumulation in response to soil drying for different crop species may allow specific areas of the root system to be dried with minimal yield penalty.

## Abbreviations:

Ψ_leaf_: leaf water potential
Ψ_root_: root water potential
*θ*_g_: soil gravimetric water content
*θ*_v_: soil volumetric water content
ABA: abscisic acid
[ABA]_root_: abscisic acid concentration in root tissue
ANOVA: analysis of variance
*g*_s_: stomatal conductance to water vapour
HPRD: horizontal partial root-zone drying
PRD: partial root-zone drying
RWU: root water uptake
RWUF: root water uptake fraction
SE: standard error
VPRD: vertical partial root-zone drying
[X-ABA],: abscisic acid concentration in xylem sap
[X-ABA]_leaf_: abscisic acid concentration in leaf xylem sap
[X-ABA]_root_: abscisic acid concentration in root xylem sap.
